# Graph configuration model based evaluation of the education-occupation match

**DOI:** 10.1371/journal.pone.0192427

**Published:** 2018-03-06

**Authors:** Laszlo Gadar, Janos Abonyi

**Affiliations:** 1 Innopod Solutions Ltd, Budapest, Hungary; 2 MTA-PE Budapest Ranking Research Group, University of Pannonia, Veszprem, Hungary; 3 MTA - PE Lendulet Complex Systems Monitoring Research Group, University of Pannonia, Veszprem, Hungary; IBM Thomas J Watson Research Center, UNITED STATES

## Abstract

To study education—occupation matchings we developed a bipartite network model of education to work transition and a graph configuration model based metric. We studied the career paths of 15 thousand Hungarian students based on the integrated database of the National Tax Administration, the National Health Insurance Fund, and the higher education information system of the Hungarian Government. A brief analysis of gender pay gap and the spatial distribution of over-education is presented to demonstrate the background of the research and the resulted open dataset. We highlighted the hierarchical and clustered structure of the career paths based on the multi-resolution analysis of the graph modularity. The results of the cluster analysis can support policymakers to fine-tune the fragmented program structure of higher education.

## Introduction

Policymakers need solid information on how labour market evaluates higher education graduates. Institutions also should collect and analyse relevant information about their graduates for the management of their programs [1]. Since the salary and the chance of finding a job are important decision factors at the college attendance [2], university and program level public information about the career paths are also important to candidates of higher education [3].

Although self-reported data can have validity problems, questionnaire based databases are useful to study education-occupation matches. Among these, the Reflex database is the most comprehensive information source in Europe. The analysis of this database showed that graduates working in the field of their study have higher income and satisfaction, so they are a happier members of the society [4].

Administrative data can replace traditional questionnaires to offer much more objective information for evidence-based educational policy in decision-making [5]. In Austria database of the whole state insurance system is accessible in anonymized form, which is also ready to career path analysis [6]. With administrative data, we can also measure the added value of higher education institutes by combining information about persistence rates, graduation rates, and post-college earnings [7]. The use of administrative data has a long tradition in Northern Europe. Finland recently connected administrative and survey data sources [8]. Based on the register of Statistics of Finland some employers were suggested to be interviewed to study unemployment of young graduates and transition from higher education to work [9]. The Swedish Ladok database was used to determine the influence of higher education institutions on labour market by regression analysis. The availability of extensive, longitudinal data made it possible to the evaluate the matching of the occupation and the level of the degree among engineering, teaching, nursing, business specialisations [10].

In our study, we try to dig deeper by focusing on the more detailed program level by proposing a goal oriented graph mining tool to evaluate the matching of programs and occupations.

In recent years, network-type models have been proven to be useful in understanding complex systems in different subject areas (e.g. sociology, economy, industry, and biology [11]). Real life entities (e.g. people, universities, educational programs) can be characterised by numerous categorical properties (e.g. education can characterise people). Relationships between entities and values of a selected property can be modelled with a two-mode network (also known as a bipartite graph) [12].

The proposed network model is based on the integration of the databases of the National Tax Administration, the National Health Insurance Fund, and the data warehouse of the Hungarian higher education. This administrative dataset covers 15 thousand people graduated in 2009/2010 school year and worked in 2012 May. Based on the data of 7402 Bachelor students we defined a bipartite graph of 110 bachelor programs and 113 occupations encoded by the third level of International Standard Classification of Occupations (ISCO) code system. The nodes of the resulted network are connected by 7400 links that represent the employees who received their bachelor level in a given program and work in a given profession. (The data will be available on the website of the author: www.abonyilab.com). To demonstrate the background of the research, we present a brief analysis of gender pay gap and the spatial distribution of over-education.

The analysis of the bipartite network shows that both the programs and the occupations follow a power law distribution which reflects there is a structure in the carrier paths. Our key idea is that we compare the weights of the edges with the expected number of edges of a random graph that has the same degrees as the studied network. This configuration model seems the most sophisticated reference because it takes into account the expected number of links by degrees of given program and occupation [13].

To search patterns in education-occupation transition in different levels of details, we cluster the graph by looking for subgraphs whose vertices are more likely to be connected to one another than to the vertices outside the subgraph [14]. To evaluate the consistency of the detected clusters we use a graph modularity based measure which assesses the quality of the clusters based on the number of edges of the configuration model [15]. We elaborated a multi-resolution type analysis of the network by the step by step removal of the weak connections. The results highlight that the educational programs have a hierarchical structure.

A large number of higher education programs can lead to a fragmented and inefficient education system. The results confirm that the extracted clusters can support decisions related to the monitoring and (re-)design of the program structure.

## Methods

### Bipartite graph model of the education to work transition

The vertices of the bipartite graph model of the education to work transition are divided into two disjoint sets, U,V. The U represents the educational programs, and the V represents the sets of occupations. Every edge connects a program to an occupation. The edges are weighted, and the weights are representing the number of graduated students in a given program connected to a specific profession. The graph can be represented by an **A** adjacency matrix, where the *A*_*ij*_ element of the matrix represents how many graduates of the *i*-th bachelor program are working on the *j*th profession.

By following this arrangement, the sum of the *i*-th row of represents the number of students graduated in the *i*-th program, while the sum of the *j*-th column represents the total number of employees having a given profession. These sums can be considered as the degree of the nodes, calculated as *k*_*i*_ = ∑_*j*_ *A*_*ij*_ and *k*_*j*_ = ∑_*i*_ *A*_*ij*_, respectively.

Not all nodes in a network have the same number of edges (same node degree). The probability that a node has < *k* > edges can be described by a distribution function *P*(*k*). The analysis of the degree distribution can show how the graduates are distributed among the programs and the occupations.

### Evaluation of the education-occupation match

To decide which education programs and occupation pairs are relevant and which can be considered as a “noisy” individual case, we propose a measurement to evaluate the strengths of the connections.

Our core idea is that we can compare the *A*_*ij*_ weight of the edge with the expected edge weight of a random graph that has the same degrees as the studied network. This configuration model, which is often referred as a random network with a pre-defined degree sequence [16], seems the most sophisticated application because it takes into account the expected number of links by degrees of given program and occupation.

If the edges were randomly distributed, $\frac{k_{i}k_{j}}{L}$ would be the expected number of links between the *i*-th program and *j*-th occupation, where *L* represents the total number of links in the network, *L* = ∑_*i*,*j*_ *A*_*ij*_, while *k*_*i*_ and *k*_*j*_ are the degrees of the program and occupation nodes, respectively [14].

Since in the case of random matching $\frac{k_{i}k_{j}}{L}$ graduates of the *i*-th program would choose the *j*-th occupation, the difference between the actual and the expected number of graduates in the case of random arrangement can be calculated as:

$$\begin{array}{c} A_{ij}-\frac{k_{i}k_{j}}{L}\; \end{array}$$ (1)

which difference can be used as a measure of the strength of the education—occupation matchings.

### Simultaneous clustering the programs and the occupations

In the previous session, we evaluated the connection of individual educational programs and occupations. To provide information about the whole structure of the network, we cluster the edges to obtain groups of similar programs and professions.

To formalise this clustering problem, we utilise the modularity measure introduced by Newman [15] and improved for bipartite graphs by Barber [14]. A module of the network is a subgraph whose vertices are more likely to be connected to one another than to the vertices outside the subgraph. Modularity reflects the extent, relative to a random configuration network, to which edges are formed within modules instead of between modules:

$$\begin{array}{c} Q=\frac{1}{L}\sum_{ij}\left(A_{ij}-\frac{k_{i}k_{j}}{L}\right)\delta \left(C_{i},C_{j}\right)\; \end{array}$$ (2)

where the Kronecker delta function *δ* is equal to one when nodes *i* and *j* are classified as being in the same module (i.e. they have the same label value) or zero otherwise.

The modularity can be determined for each community of a network. A network with *n*_*c*_ communities, the following modularity value is used to determine *M*_*c*_ community modularity value. Each *C*_*c*_ community with *N*_*c*_ nodes are connected with by *L*_*c*_ links, *c* = 1, … *n*_*c*_.

$$\begin{array}{c} M_{c}=\frac{1}{L}\sum_{\left(i,j\right)\in C_{c}}\left(A_{ij}-\frac{k_{i}k_{j}}{L}\right) \end{array}$$ (3)

The *M*_*c*_ modularity value of a *c* cluster can be either positive, negative or zero. In the case of zero, the community has as many links as a random subgraph. If it is a positive value, then the *C*_*c*_ subgraph tend to be a community, while a negative *M*_*c*_ means it is not.

We used the multi-level modularity optimization algorithm (so called Louvain algorithm) to find clusters in the programs-occupation bipartite graph. This algorithm uses an iterative procedure to assign each node to a module by maximising the modularity [17].

The rows and the columns of the adjacency matrix of the bipartite graph can be reordered to visualise the similarities of the programs and relationships (see later in chapter Clustering and Visualization).

### Multi-resolution cluster analysis

Since we would like to determine how the significant matchings are structured, we applied a method for cleaning the network by step by step removing of the weak connections.

$$\begin{array}{c} {\tilde{A}}_{ij}=\{\begin{array}{cc} A_{ij}, & if\;A_{ij}\geq \frac{k_{i}k_{j}}{L}\cdot \alpha \\ 0, & if\;A_{ij}<\frac{k_{i}k_{j}}{L}\cdot \alpha \end{array} \end{array}$$ (4)

As the Eq 4 describes the cleaning procedure has one 0 ≤ *α* threshold parameter. When *α* = 0, none of the edges are removed. It should be noted, that *α* can be considered as a minimum relative edge strength. After the pruning of network, all connections will have *α* times larger weight than weight we would expect based on the random configuration model:

$$\begin{array}{c} \frac{{\tilde{A}}_{ij}}{\frac{k_{i}k_{j}}{L}}\geq \alpha \;\forall i,j \end{array}$$ (5)

It should be noted that this equation measures how the given edge contributes to the Louvain ratio used to measure the compactness of a module/cluster:

$$\begin{array}{c} LR_{C_{c}}=\frac{A_{C_{c}}}{P_{C_{c}}}\;,\;A_{C_{c}}=\sum_{\left(i,j\right)\in C_{c}}A_{ij}\;,\;P_{C_{c}}=\sum_{\left(i,j\right)\in C_{c}}\frac{k_{i}k_{j}}{L}\;. \end{array}$$ (6)

As can be seen later in chapter Evaluation of Education—Occupation Matching, it is interesting to analyse how the step-by step increase of this parameter decreases the network density, what is the ratio of the non-significant edges, how characteristically structured the network.

Since after this pruning we also applied modularity based clustering, the resulted method can be considered as a special multi-resolution analysis technique.

Modularity optimization based community detection has a resolution limit, failing to detect communities smaller than a scale that depends on the total number of edges in the network and degree of interconnectedness of the communities [18]. To handle this problem multi-resolution methods were introduced by adjusting the resolution of the algorithms by modifying the modularity function, weighting the contribution of the null model [19] or adding self-loops to the nodes [20]. These methods still have the intrinsic limitations that large communities may have been split before small communities become visible [21].

Since the problem is that modularity based community detection algorithms join small fully connected subgraphs connected only by weak edges into larger groups [22], our methodology which gradually removes the less important connections by the increase of *α* can also be considered a graph-modification based multi-resolution approach that handles this problem. It should be noted, that we developed our algorithm as we were interested in how the statistically significant education-occupation matchings are structured.

## Results and discussion

### Administrative data of the Hungarian career path tracking system

The development of our methodology is motivated by the question, how the Hungarian carrier path tracking system can be used to support evidence based policy making and monitoring.

The studied administrative government data were collected as the integration the databases of the Hungarian tax office and National Health Insurance Fund in 2014. The database was designed by using individual hash codes, so although it does not contain personal information. It allows the micro data level analysis of student career paths.

The integrated database contains 70 variables about

- personal data (date of birth, county of address, citizenship, gender)
- occupational data in the year of 2012 (employment relationship, occupation, gross wage, etc.)
- employer data (county of company headquarters, company size, company activity, etc.)
- if the graduate runs her/his own enterprise the primary data of the company
- educational data (institute, faculty, program where the graduate graduated)

The dataset contains 29873 individual records. Among these only 15253 people have occupational data [23]. It must be noted, that this integration was the first made by the related governmental organisations in Hungary, so probably this is the reason why only the half of the persons were correctly merged. Table 1 shows contents of published database. The correctly identified 15253 people graduated 398 education programs delivered by 52 institutions and worked in 402 occupations encoded by the fourth level (unit groups) of the International Standard Classification of Occupations (ISCO) code system. In this work, we focus on just bachelor degree graduates. Among 15253 people 7402 has a bachelor degree in 45 institutions, 110 programs, and works in 372 occupations, and 113 third level occupation groups. This database is open for research purposes. The cleaned dataset used in this study is available on our website: www.abonyilab.com

**Table 1:** Variables of the dataset.

Column name	Description
ID	ID of the graduate
Gender	Gender
Inst_HUN	Institution name in Hungarian
Pr_area_HUN	Training program areas in Hungarian
Pr_HUN	Training programs in Hungarian
Pr_ENG_bachelor	Training programs of bachelors in English
Grad_level	Degree levels
FEOR4	4th level of Hungarian ISCO
FEOR3	3rd level of Hungarian ISCO
ISCO1	1st level of ISCO
ISCO3	3rd level of ISCO
Req_HEd	Jobs that does require HE degree
Head_couty_HUN	Couty of company headquarter
Weekly_hrs	Weekly working hours
Mountly_wage_HUF	Mountly gross wage in HUF

### Measuring overeducation and gender pay gap

Interesting point of the dataset that according to the main group of ISCO code we can determine that a given occupation requires higher education degree or not. Table 2 shows how much percentage of the graduates has jobs that require higher education degree.

**Table 2:** Distribution of graduates working in occupation category that requires higher education degree (HEd).

Education level	Require HEd	Not HEd	Number of graduates
Higher vocational trainings	38.9%	61.1%	921
Bachelor	68.8%	31.2%	7402
Collage (equal with Bachelor)	66.9%	33.1%	1889
Master	90.5%	9.5%	1334
University (equal to Master)	84.8%	15.2%	3252
Special teacher programs	67.5%	32.5%	409

**Fig 1:**
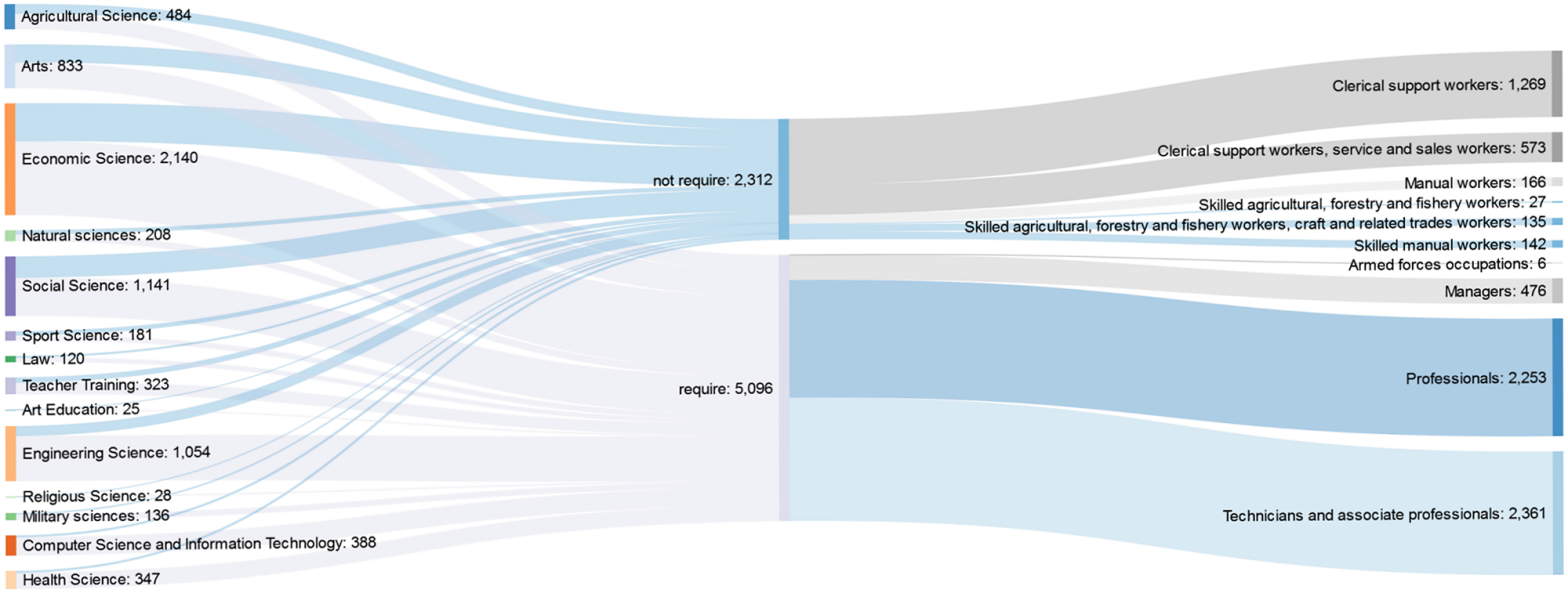
Distribution of bachelor graduates working in an occupation that requires higher education degree.

The Shankey diagram of the BSc/BA graduates (see Fig 1) shows that who graduated in computer science and information technology, health science, engineering science works more likely in an occupation that requires higher education degree compared with graduates in sports science, arts and humanities, natural sciences, agricultural science.

Working in a field that matches to the education has a positive effect on job performance and satisfaction [4]. Results of Iriondo and Pérez-Amaral indicates that overeducated workers suffer a wage penalty since earnings depend mainly on the educational requirements of jobs [24]. Three primary measures of education—job mismatch can be distinguished based on how the required education level is determined. The first method relies on the self-assessment [25], the second approach evaluates the “realised matches” [26], while the third “job analysis method” refers to a systematic evaluation of the “professional job analysts” who specify the required level of education for the job titles in an occupational classification [27] [28]. These last two methods require large scale and up to date administrative data-based studies, similar that we would like to deliver in our research.

Groot and Maassen van den Brink conducted a meta-analysis of 25 studies on overeducation and found that the matching based methods show 13.1% of overeducation, while self-assessment based studies estimate the much higher percentage of overeducated employees, 28.6% [29]. McGuiness and Sloane used the REFLEX dataset to study overeducation in the UK. When both education and skill mismatch variables were included in the model, overskilling reduced job satisfaction consistently for both sexes. In the UK 36% of the respondents felt as overeducated, which is quite high, compared to the 14% measured elsewhere in Europe [30], and 17% in Taiwan in 2008 [31].

A much more objective study has been performed in Spain where the Spanish Wage Structure Survey (WSS) dataset was used to examine the effects of educational mismatch on wages. Based on this employer-worker microdata 32-37% overeducation rates were calculated [32].

**Fig 2:**
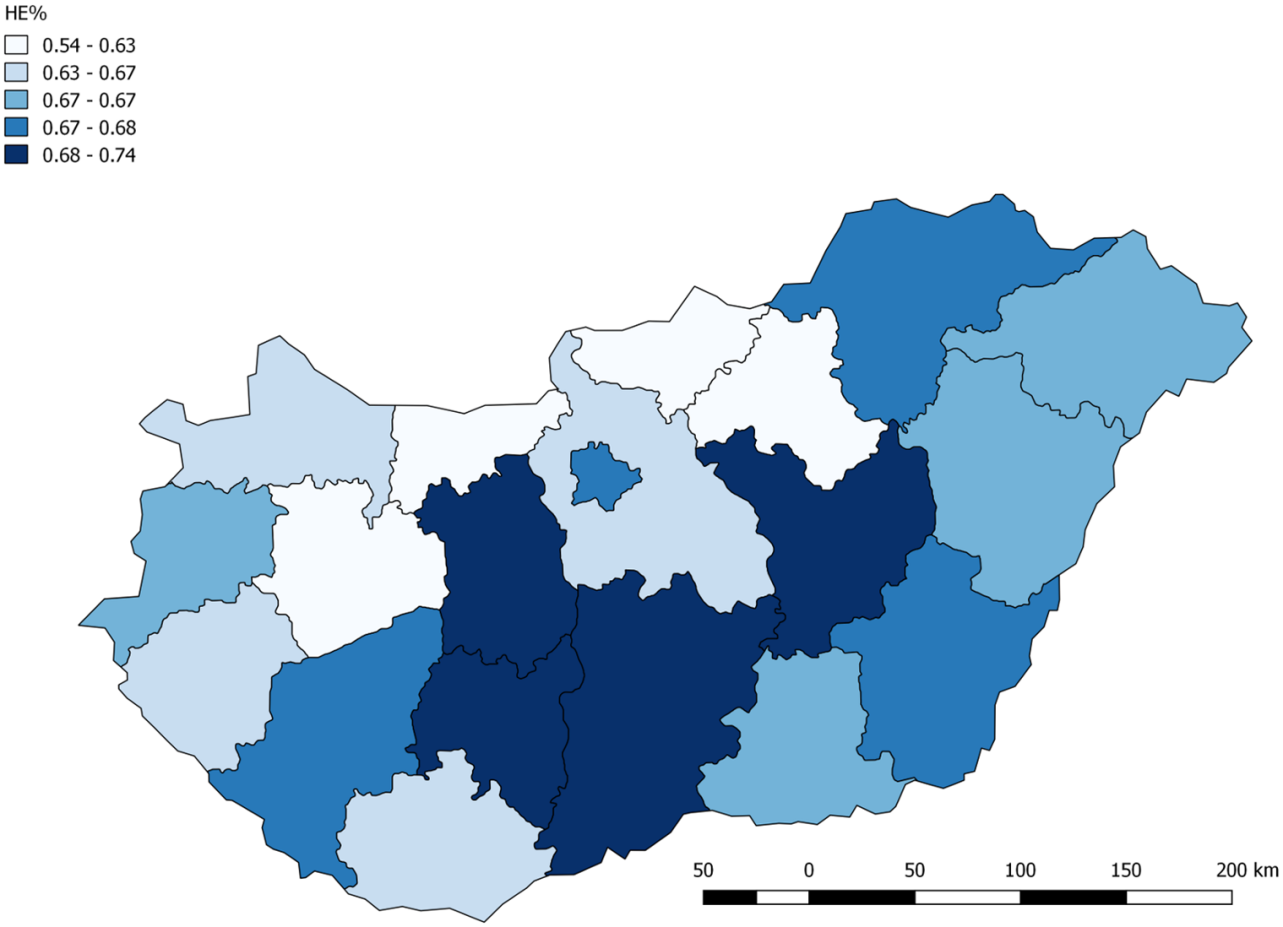
Distribution of graduates that work on occupation which requiring higher education degree by counties in Hungary.

Our database allows a more detailed analysis. Similarly to other countries, this dataset also shows 26-39% of overeducated employees. The spatial distribution of occupations of the graduates was also investigated. Fig 2 shows how much percentage of the bachelor graduates are working in jobs that require higher education. This figure well illustrates that the problem of over-education is mainly related to the economic development of the regions.

**Fig 3:**
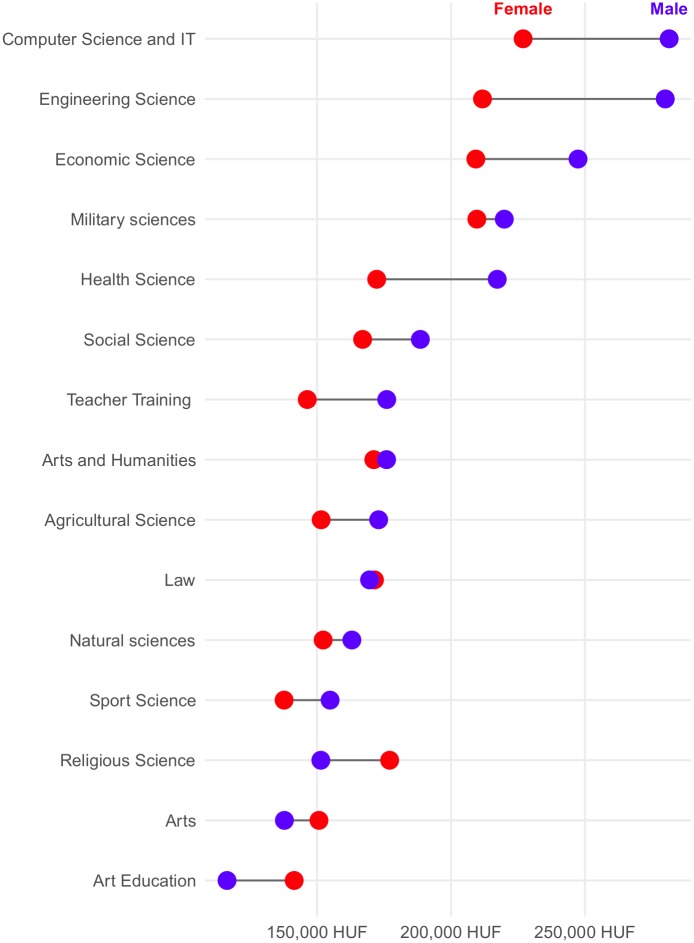
Gender pay gap grouped by education program areas.

**Fig 4:**
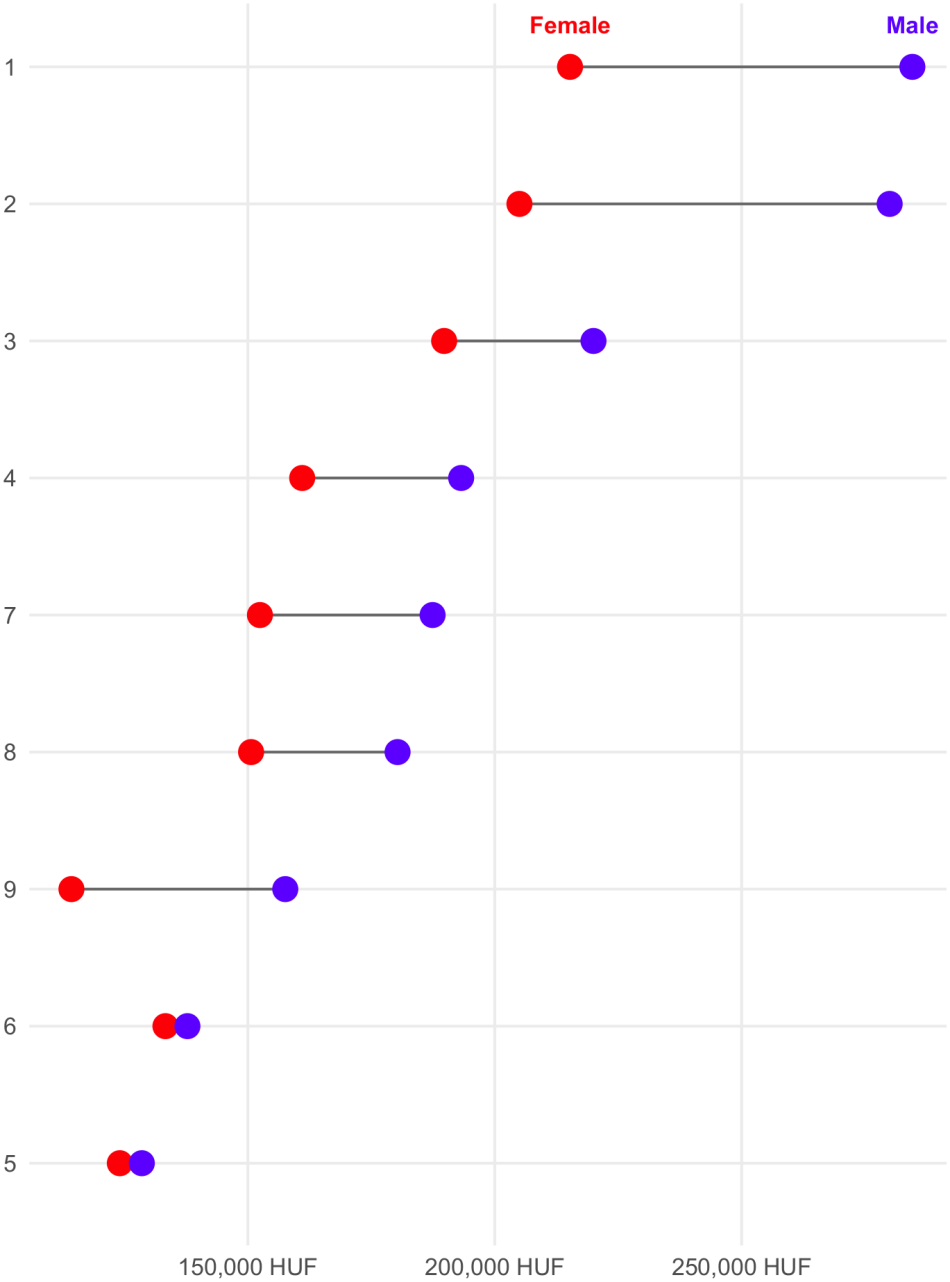
Gender pay gap grouped by occupations (first level ISCO).

The database also allows the sophisticated analysis of the gender pay gap. Fig 3 shows income gaps grouped by different education areas and Fig 4 shows incomes of different occupation categories according to the first level ISCO.

### Evaluation of the degree distributions

**Fig 5:**
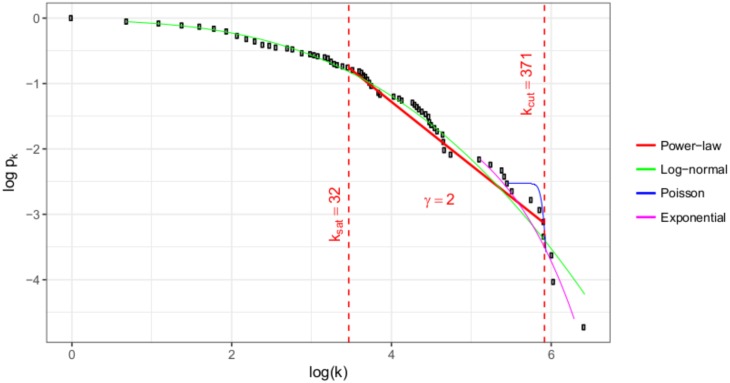
Distribution of the weighted degrees of the occupations.

**Fig 6:**
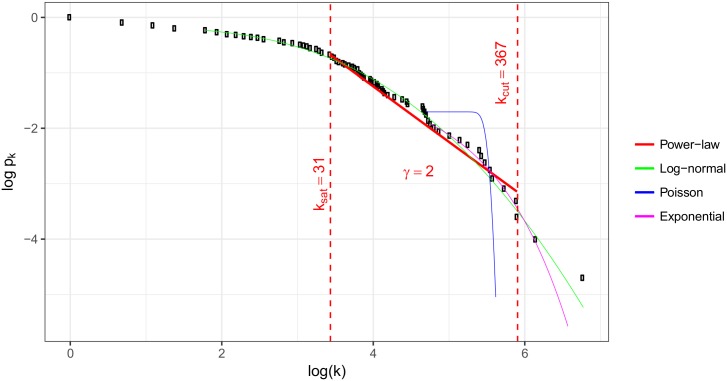
Distribution of the weighted degrees of the bachelor programs.

The cumulative degree distributions of the bipartite network are shown in Figs 5 and 6. The *k*_*i*_ weighted degrees of the five biggest programs are: Business Management: 874, Communication and Media Science: 469, Andragogy: 367, Tourism: 364, Finance and Accounting: 310. The *k*_*j*_ degrees of the top five occupations are: Business services agents: 614, financial and mathematical associate professionals: 419, Engineering professionals (excluding electrotechnology): 410, Legal professionals: 372, Sales and purchasing agents and brokers: 371.

To evaluate the whole structure of the network we fitted power-law distribution, exponential, Poisson, log-normal distributions to degrees of nodes and occupations in R with the help of the *poweRlaw package* [33].

As can be seen, there is a well defined linear region between *k*_*sat*_ and *k*_*cut*_. The slope of this linear trend gives *γ* which is 2.00. There are less number of small degree nodes (occupations) then power-law fit would require therefore in *k* < *k*_*sat*_ region data point are below the extrapolation of fitted line. Similarly, there are less number of high degree nodes or hubs then power-law fit would require thus in *k*_*cut*_ < *k* region data point are also below the extrapolation of fitted line.

The degree distribution of these scale free networks can be described by a power-law tail function, *P*(*k*) = *k*^−*γ*^ [34], and the *γ* parameter is one of the most important property of a graph.

The question is that which model is closer to the empirical distribution. The p-values shown in Table 3 suggest that we cannot reject the null hypothesis that the data follows power-law distribution.

**Table 3:** Results of fitting power-law to bipartite graph.

Graph set	*K*_*min*_	*K*_*max*_	*k*_*sat*_	*k*_*cut*_	D	p	*γ*
Programs	1	875	31	367	0.084	0.93	2.00
Occupations	1	614	32	371	0.101	0.87	2.00

Power-law and log-normal distributions were compared using Vuong’s test statistic [35]. The two sided p-value of comparison test shows that both distributions are equally close to the empirical distribution.

When the nodes of a network are randomly connected, *γ* is bigger than three. 2 < *γ* < 3 relates to scale-free networks [16]. Our analysis shows that the studied bipartite network has scale-free structure, which proves that graduates do not randomly choose an occupation, and they preferences can be studied by a more detailed analysis of the edges.

### Evaluation of education—Occupation matching

The proposed graph configuration model based measure can be used for ranking the education—occupation matchings. Tables 4 and 5 show the strongest and the weakest educational program—occupation pairs.

**Table 4:** Top 10 strongest connection.

Programs	Professions	# links	# links (conf. model)
Business Management	Financial and mathematical profess.	153	49
Mechanical Engineer	Engineering professionals	114	14
Engineering IT Specialist	Software and applications developers	102	3
Tourism and Hospitality	Client information workers	83	17
Finance and Accounting	Financial and mathematical associate	80	18
Electrical engineering	Electrotechnology engineers	65	3
Nursing and Patient Care	Other health professionals	55	2
Business Management	Numerical and material recording clerks	58	10
Business Management	Business services agents	84	36
Nursing and Patient Care	Nurses and midwives	47	2

**Table 5:** Top 10 weakest connection.

Programs	Professions	# links	# links (conf. model)
Tourism and hospitality	Engineering professionals	1	20
Electrical engineering	Business services agents	3	22
Communication and Media Science	Engineering professionals	6	26
Finance and Accounting	Client information workers	1	14
Electrical engineering	Financial and mathematical associate	1	14
Andragogy	Engineering professionals	4	20
Finance and Accounting	Engineering professionals	3	18
Commerce and Marketing	Engineering professionals	1	14
Business Management	Software and applications developers	9	28
Communication and Media Science	Software and applications developers	2	14

**Fig 7:**
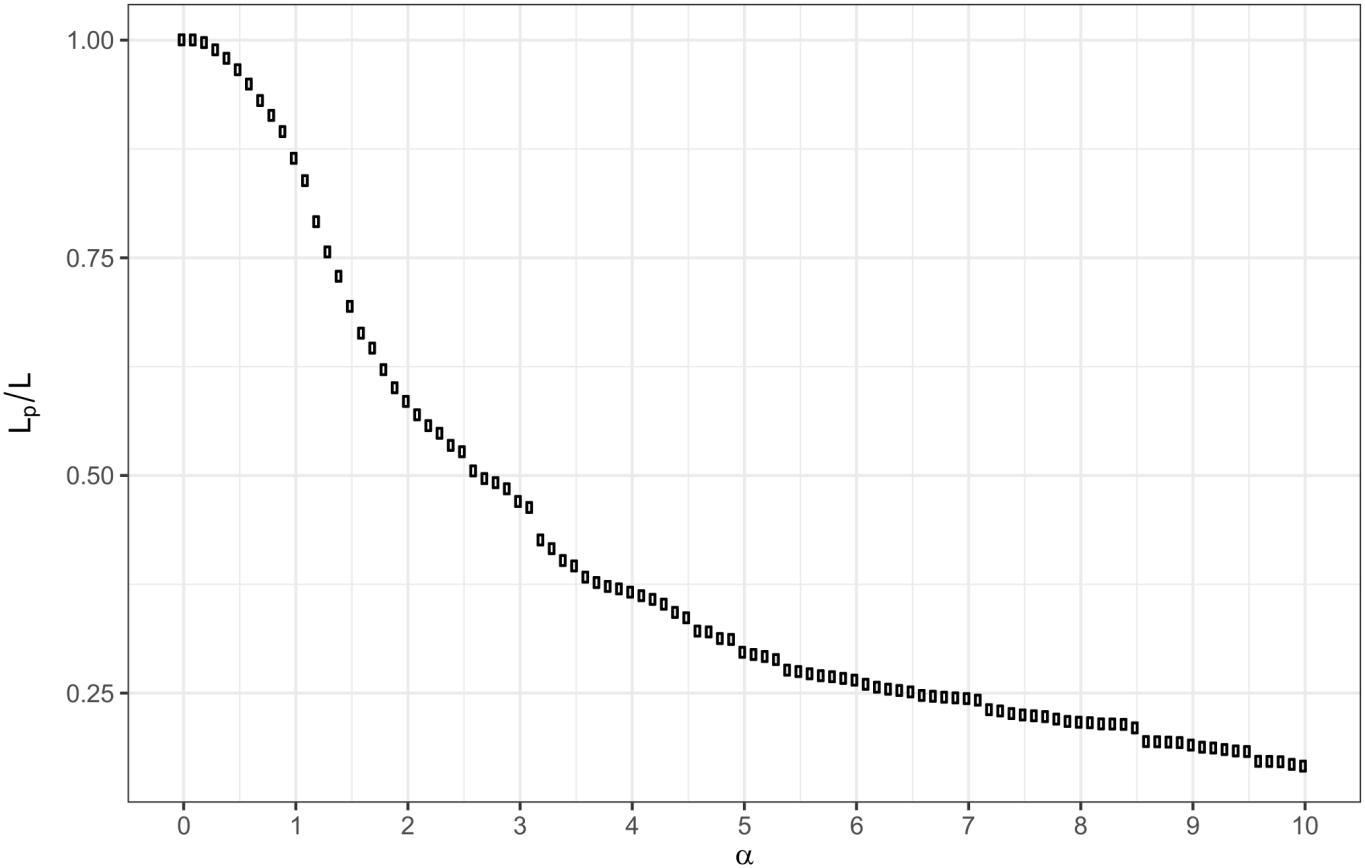
Distribution of the education—occupation significance values as the ratio of the remaining edges after pruning with different *α*.

Fig 7 shows the distribution of the weak edges that will be removed at a given *α* threshold.

### Degree correlation and centrality measures

Programs with a relatively small number of connections are not “spread”. In some of these programs, there are a lot of graduates, but they work in a few kind of occupations. These programs are the following: computer science, engineering (electrical engineer, mechanical engineer, civil engineer, chemical engineer, mechatronic engineer), special need teacher, nursing and patient care, laboratory and diagnostic imaging analyst, and economic science (finance and accounting, international management, management organisation). Conversely, only small number of agricultural, teacher training, liberal arts, light industrial engineering graduates work in several kinds of occupation.

To get more information about the structure of our network, we calculated the degree correlation as the correlation of the node degree with the node degree of the neighbour nodes. The results show that our graph is disassortative, which means high-degree components (hubs) tend to connect to low-degree nodes, while low-degree nodes are connected to hubs [16]. In practice, this means that graduates of programs on that few people graduated work in popular occupations.

Computer science, nursing, and engineering programs are interesting in the context of eigenvector centrality as well. These programs are mainly connected to occupations that have few kind of “supplier”, so they are not so embedded in the graph. This group of programs have relatively high betweenness and low closeness centrality which means that they include many shortest path, but they are far from the centre of the graph. This phenomenon predicts that the related programs—occupations connections are strong and form subgraphs that have fewer connections to other parts of the graph.

### Clustering and visualization

**Fig 8:**
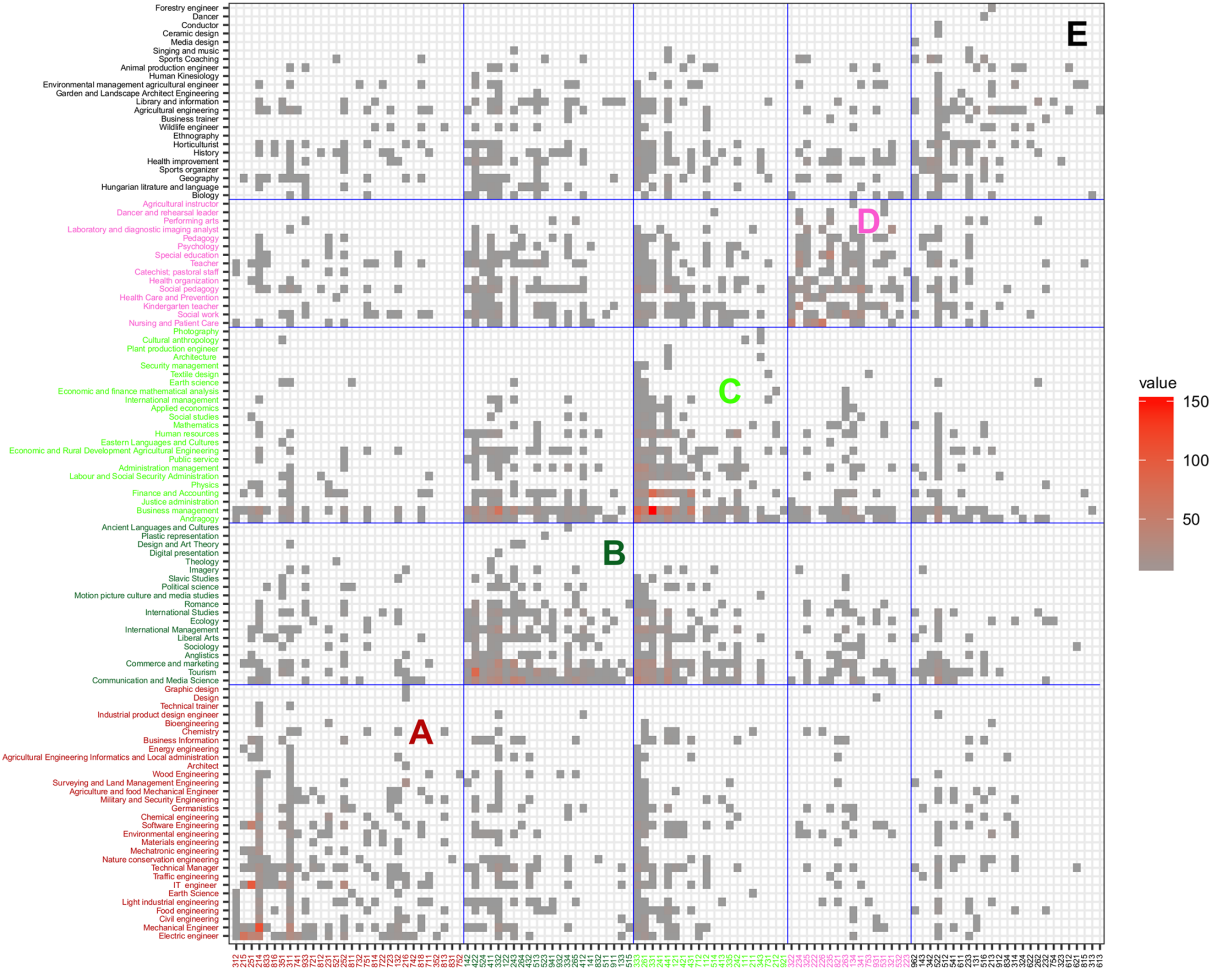
The modules obtained by the Louvain algorithm of purified program/occupation bipartite graph.

During clustering each program and occupation were assigned to one module, so each module contains a set of programs and professions. The result of clustering can be seen in Fig 8. This figure shows the adjacency matrix with columns and rows ordered according to the result of the clustering. The resulting five clusters are marked by A-E letters are the diagonal blocks of this matrix. Table 6 shows the Louvain ratio (see Eq 6) for every module.

**Table 6:** Louvain ratios of the pairs of progam-occupation clusters.

	A	B	C	D	E
E	0.614	0.860	0.584	0.666	**4.152**
D	0.246	0.664	0.498	**4.051**	0.694
C	0.209	0.829	**1.826**	0.429	0.572
B	0.225	**2.010**	0.962	0.348	0.797
A	**3.200**	0.430	0.370	0.208	0.556

Module ‘A’ consists of engineering programs supplemented with design, technical trainer and germanistics programs. This cluster highlights that 25% of graduates of germanistics work in manufacturing and IT sector, indicating the strong presence of the German industry requiring advanced knowledge of German language. Module ‘B’ consists of economy, social, political, and language teacher programs weakly connected to sales, office workers, client information, journalist, brokers, marketing and PR professionals. Module ‘C’ contains management, financial economic, and agricultural programs, along with small programs such as physics, earth science, cultural anthropology. With them, financial, business, clerk, trade workers, keyboard operators, and service worker occupations are associated. It should be noted that there is a relatively strong connection between the ‘B’ and ‘C’ modules. Module ‘D’ connects medical, pedagogy, teacher, social work, dancer and arts type programs with health, teaching, child care workers, medical technicians, and personal care workers. In this module, there are the fewest number of occupations which do not require higher education diploma. Module ‘E’ collects programs with a small number of graduates. This module shows how agricultural, natural science, teacher, sport, art type of programs are connected to operators, technicians, workers, vocational education teacher, animal producer, crop grower, cooks, salesperson, services manager, and life science professionals.

In the case of the A, D and E modules this ratio shows a larger difference from the null model compared to the B and C models, which indicates the stronger connection of programs and occupation in the A, D, and E modules.

During our work, we tested several clustering algorithms, including the BRIM algorithm (bipartite, recursively induced modules) developed specifically for bipartite graphs [14].

**Fig 9:**
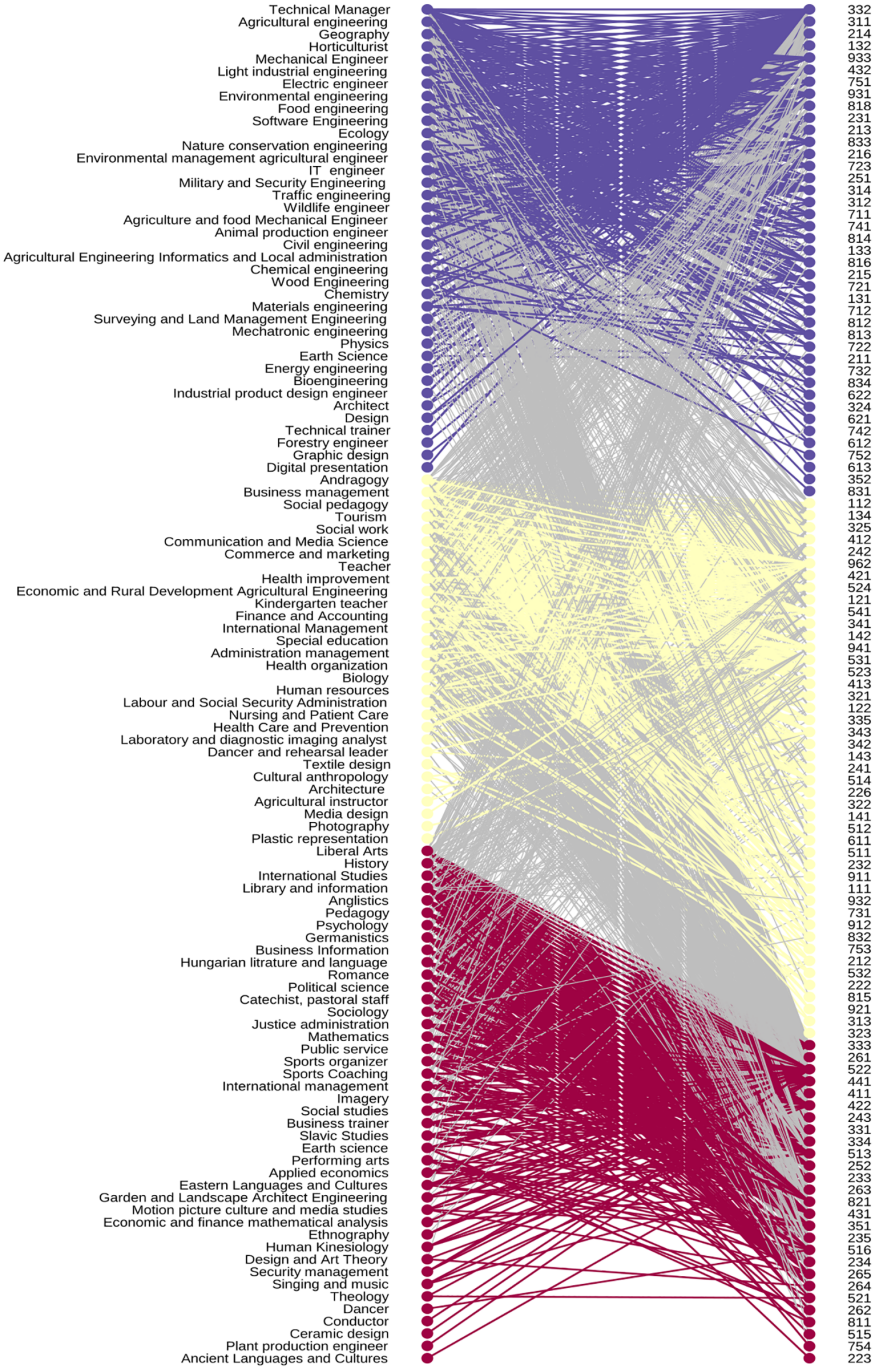
Clustering and reordering of the bipartite graph with Barber algorithm.

As Fig 9 shows, the first module contains mainly teaching, humanity and art programs. The second module exhibit business, economic, finance, HR, social work, nursing, medical programs. In this module, almost half of the occupations do not require higher education diploma, like cooks, hairdresser, personal services, cashier, personal care, food preparation assistant, elementary worker.

The third module represents natural and technical sciences programs, like engineering, IT, physics, ecology, earth science connected to production, manufacturing, information managers, life science, engineering professionals.

### Application of multi-resolution cluster analysis

**Fig 10:**
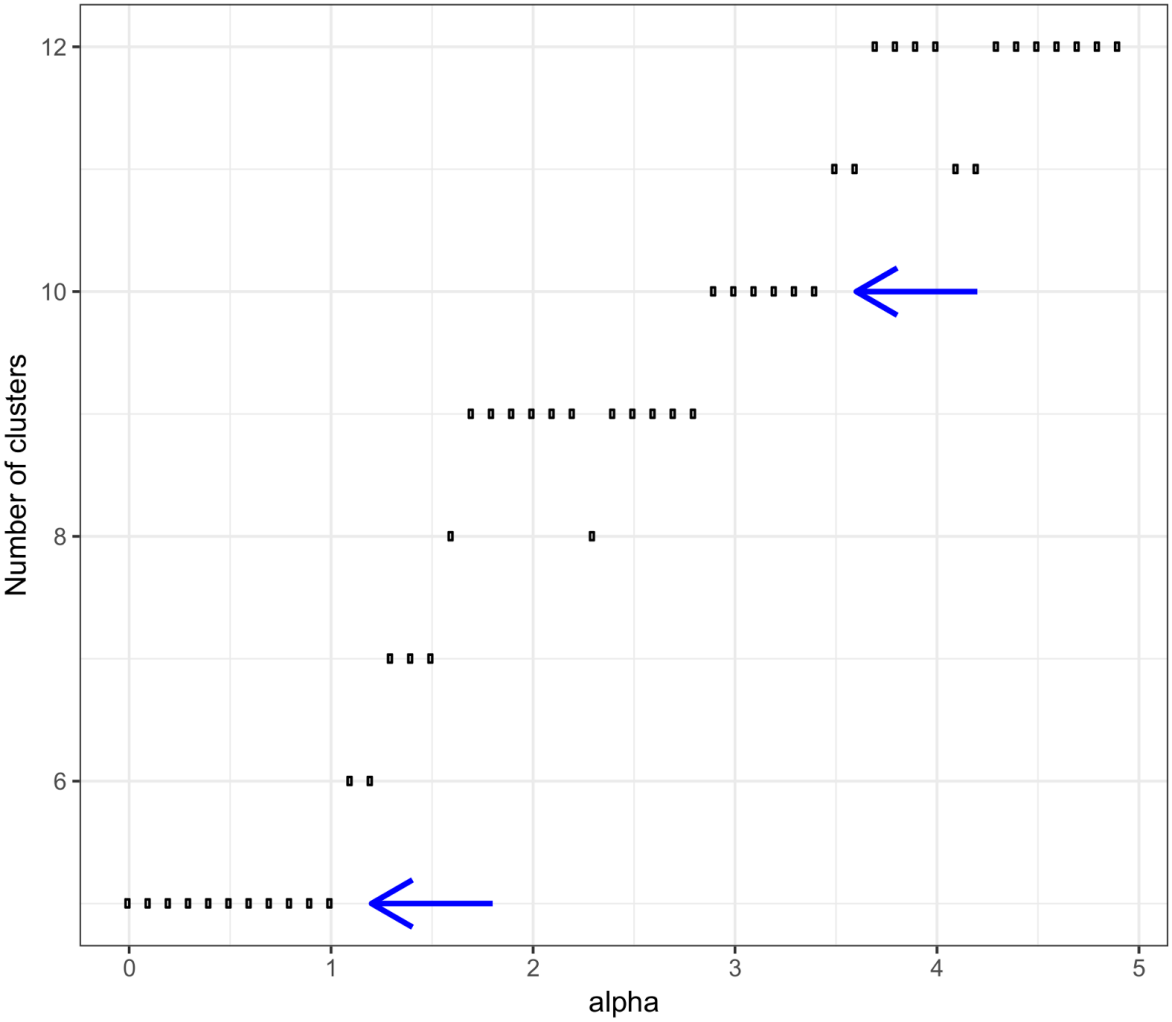
Number of clusters in case of different *α*.

Louvain modularity optimization algorithm was performed with different *α*. Fig 10. shows the number of clusters resulted from Louvain algorithm in the function of *α*.

We detected hierarchical structure since all communities found at a value of *a*_2_ > *a*_1_ are sub-communities of the communities found at *a*_1_. We measured the similarities of the nodes based on whether their share the same cluster in different resolutions. A hierarchical splitting occurs when the cluster of health type programs splits into nursing and medical professionals. Similarly, the cluster of pedagogy and social programs is divided into teaching and social professionals and the group of child care workers.

**Fig 11:**
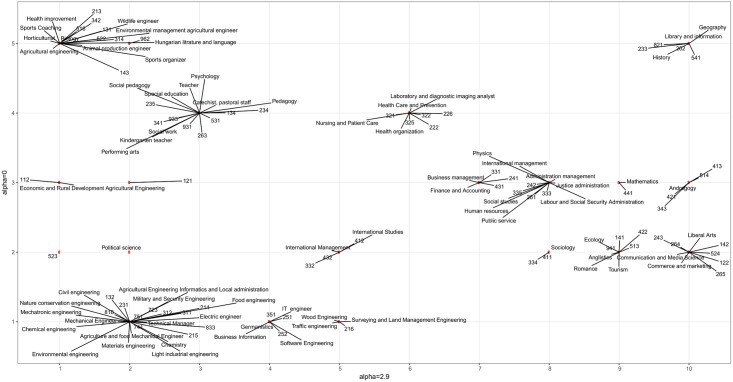
Relationship of clusters generated in step one and step three of the multi-resolution analysis.

The relationships of the clusterings resulted in different resolution level is visualised in Fig 11.

As can be seen, somewhat hierarchical splitting occurred in the first cluster. By increasing *α* IT engineer, business information, software engineering, germanistics programs with software developer, database professionals, information technology operations technicians occupations separated (see Fig 11 *y* = 1, *x* = 3)

## Conclusion

Administrative data based career path analysis can of support governmental policy making and program development of higher education institutes. To support the extraction of useful information from these databases we developed a graph-based data structure to represent the career path of higher education graduates. Education—occupation mismatch can be analysed based on the bipartite graph of bachelor programs and occupations encoded by International Standard Classification of Occupations (ISCO) code system. We modified the Newman modularity measure to evaluate the matching of the programs and the professions. Based on this measure the hidden structure of career paths can also be clustered and visualised.

The proposed network model is applied on the integrated databases of the National Tax Administration, the National Health Insurance Fund, and the data warehouse of the Hungarian higher education. To demonstrate the information content of this administrative database, we presented a brief analysis of the gender pay gap and the spatial distribution of the over-education. Similarly to other countries, we showed 26-39% of overeducated employees.

The transition of graduates from higher education to employment is affected by individual characteristics. However, graduates with well-defined qualification start working in a somewhat similar profession. Our graph model gives the opportunity to cluster the typical career paths and find outliers whose education and occupation does not match. The results illustrate that the proposed multi-respolution type community finding approach provides useful results, as it highlights the groups of programs that are strongly connected to groups of bachelor programs.

The analysis of the clusters allows us the more sophisticated analysis of the performances of the programs in the labour market. For example, our method showed that significant proportion of graduates of Germanistics work as a system administrator with approximately 30% higher salary indicating the strong presence of German origin industry in Hungary.

Such results can be useful for education policy experts and decision makers who can see the structure of the Bachelor programs from the objective viewpoint of the labour market. The resulted orderings and matching measures can support the policymakers to fine-tune the fragmented program structure of the Hungarian higher education. We found bachelor programs that are almost identical in their content, but they are different from the view of the labour market because graduates work in the different occupation. For example, pedagogy and andragogy use similar methods, but the graduates of andragogy work profession that is more related to communication and media science. (Probably this was one of the reasons why the andragogy Bachelor program has been closed in Hungary in 2017.)

The results are also informative to students and applicants of the higher education who want to be prepared for a finding job with good expectations.

## References

[1] ENQA. Standards and Guidelines for Quality Assurance in the European Higher Education Area (ESG). 2015; p. 32.

[2] Bogdan Sojkin S A Bartkowiak Paweł. Determinants of higher education choices and student satisfaction: The case of Poland. Higher Education. 2012;63(5):565–581. doi: 10.1007/s10734-011-9459-2

[3] Cremonini L, Westerheijden D, Enders J. Disseminating the right information to the right audience: Cultural determinants in the use (and misuse) of rankings. Higher Education. 2008;55(3):373–385. doi: 10.1007/s10734-007-9062-8

[4] García-Aracil A. Effects of college programme characteristics on graduates’ performance. Higher Education. 2015;69(5):735–757. doi: 10.1007/s10734-014-9803-4

[5] Wallgren A, Wallgren B. To understand the Possibilities of Administrative Data you must change your Statistical Paradigm! In: Joint Statistical Meetings. Section on Survey Research Methods; 2011. p. 357–365.

[6] Gál A. Adminisztratív adatok pályakövetési célú felhasználásának nemzetközi gyakorlata (in Hungarian). In: Garai O, Veroszta Z, editors. Államigazgatási adatbázisok a diplomás pályakövetésben; 2013. p. 5–46.

[7] Cunha JM, Miller T. Measuring value-added in higher education: Possibilities and limitations in the use of administrative data. Economics of Education Review. 2014;42:64–77. doi: 10.1016/j.econedurev.2014.06.001

[8] Garam I. Study on the relevance of international student mobility to work and employment. Finnish Employers’ View on Benefits of Studying and Work Placement Abroad Helsinki: Centre for International Mobility. 2005;.

[9] Saar E, Unt M, Kogan I. Transition from educational system to labour market in the European Union a comparison between new and old members. International journal of comparative sociology. 2008;49(1):31–59. doi: 10.1177/0020715207088586

[10] Berggren C. The Influence of Higher education institutions on labor market outcomes. European Education. 2010;42(1):61–75. doi: 10.2753/EUE1056-4934420103

[11] Young D, Borland R, Coghill K. An Actor-Network Theory Analysis of Policy Innovation for Smoke-Free Places: Understanding Change in Complex Systems. Am J Public Health. 2010;100:1208–1207. doi: 10.2105/AJPH.2009.184705 20466949 10.2105/AJPH.2009.184705 PMC2882392

[12] Morris S, Yen GG. Construction of bipartite and unipartite weighted networks from collections of journal papers. arXiv preprint physics/0503061. 2005;.

[13] Barabási AL. 2.7. In: Network Science Graph Theory. Creative Commons: CC BY-NC-SA 2.0; 2014.

[14] Barber MJ. Modularity and community detection in bipartite networks. Physical Review E—Statistical, Nonlinear, and Soft Matter Physics. 2007;76(6):1–11. 10.1103/PhysRevE.76.066102 18233893

[15] Newman MEJ. Modularity and community structure in networks. Proceedings of the National Academy of Sciences of the United States of America. 2006;103(23):8577–8582. doi: 10.1073/pnas.0601602103 16723398 10.1073/pnas.0601602103 PMC1482622

[16] Barabási AL. Network science; 2015.

[17] Blondel VD, Guillaume JL, Lambiotte R, Lefebvre E. Fast unfolding of communities in large networks. Journal of Statistical Mechanics: Theory and Experiment. 2008;10008(10):6.

[18] Fortunato S, Barthélemy M. Resolution limit in community detection. Proceedings of the National Academy of Sciences. 2007;104(1):36–41. doi: 10.1073/pnas.0605965104 10.1073/pnas.0605965104 PMC1765466 17190818

[19] Reichardt J, Bornholdt S. Statistical mechanics of community detection. Physical Review E. 2006;74(1):016110 doi: 10.1103/PhysRevE.74.016110 10.1103/PhysRevE.74.016110 16907154

[20] Arenas A, Fernandez A, Gomez S. Analysis of the structure of complex networks at different resolution levels. New Journal of Physics. 2008;10(5):053039 doi: 10.1088/1367-2630/10/5/053039

[21] Kumpula JM, Saramäki J, Kaski K, Kertész J. Limited resolution and multiresolution methods in complex network community detection. Fluctuation and Noise Letters. 2007;7(03):L209–L214. doi: 10.1142/S0219477507003854

[22] Xiang J, Hu K. Limitation of multi-resolution methods in community detection. Physica A: Statistical Mechanics and its Applications. 2012;391(20):4995–5003. doi: 10.1016/j.physa.2012.05.006

[23] Nyüsti S, Veroszta Z. Diplomás pályakövetési adatok 2013 Adminisztratív adatbázisok integrációja (in Hungarian); 2013.

[24] Iriondo I, Pérez-Amaral T. The effect of educational mismatch on wages in Europe. Journal of Policy Modeling. 2016;38(2):304–323. doi: 10.1016/j.jpolmod.2015.12.008

[25] Duncan GJ, Hoffman SD. The incidence and wage effects of overeducation. Economics of Education Review. 1981;1(1):75–86. doi: 10.1016/0272-7757(81)90028-5

[26] Verdugo RR, Verdugo NT. The impact of surplus schooling on earnings: Some additional findings. Journal of Human Resources. 1989; p. 629–643. doi: 10.2307/145998

[27] Hartog J. Over-education and earnings: where are we, where should we go? Economics of education review. 2000;19(2):131–147. doi: 10.1016/S0272-7757(99)00050-3

[28] Kler P. Graduate overeducation in Australia: A comparison of the mean and objective methods. Education Economics. 2005;13(1):47–72. doi: 10.1080/0964529042000325207

[29] Groot W, Van Den Brink HM. Overeducation in the labor market: a meta-analysis. Economics of education review. 2000;19(2):149–158. doi: 10.1016/S0272-7757(99)00057-6

[30] McGuinness S, Sloane PJ. Labour market mismatch among UK graduates: An analysis using REFLEX data. Economics of Education Review. 2011;30(1):130–145. doi: 10.1016/j.econedurev.2010.07.006

[31] Hung CY. Overeducation and undereducation in Taiwan. Journal of Asian Economics. 2008;19(2):125–137. doi: 10.1016/j.asieco.2008.02.001

[32] Murillo IP, Rahona-López M, Salinas-Jiménez MdM. Effects of educational mismatch on private returns to education: An analysis of the Spanish case (1995-2006). Journal of Policy Modeling. 2012;34(5):646–659. doi: 10.1016/j.jpolmod.2011.07.012

[33] Gillespie CS. Fitting Heavy Tailed Distributions: The poweRlaw Package. Journal of Statistical Software. 2015;64(2):1–16. doi: 10.18637/jss.v064.i02

[34] Albert R, Barabási AL. Statistical mechanics of complex networks. Reviews of Modern Physics. 2002;74(1):47–97. doi: 10.1103/RevModPhys.74.47

[35] Vuong QH. Likelihood ratio tests for model selection and non-nested hypotheses. Econometrica: Journal of the Econometric Society. 1989; p. 307–333. doi: 10.2307/1912557

